# Effect of the Scale-Up of Dolutegravir on Retention in Care, Risk of Developing Tuberculosis and Viral Load Suppression Among People Living With HIV: Analysis of Routine HIV Clinical Data in Rural KwaZulu-Natal, South Africa (2019–23)

**DOI:** 10.1093/ofid/ofag156

**Published:** 2026-03-19

**Authors:** Reuben Christopher Moyo, Elphas Okango, Larisse Bolton, Margot Otto, Ntombifuthi Blose, Tumelo Sereo, Letoao Shoeshoe, Wilfred Otambo, Dickman Gareta, Peter S Nyasulu, Paul Mee, Richard J Lessells, Frank Courteney Tanser

**Affiliations:** South African Centre for Epidemiological Modelling and Analysis (SACEMA), Centre for Epidemic Response and Innovation (CERI), School for Data Science and Computational Thinking, Stellenbosch University, Stellenbosch, South Africa; Department of Global Health, Division of Epidemiology and Biostatistics, Faculty of Medicine and Health Sciences, Stellenbosch University, Cape Town, South Africa; Africa Health Research Institute, Durban, South Africa; South African Centre for Epidemiological Modelling and Analysis (SACEMA), Centre for Epidemic Response and Innovation (CERI), School for Data Science and Computational Thinking, Stellenbosch University, Stellenbosch, South Africa; Department of Paediatrics and Child Health, Faculty of Medicine and Health Sciences, Stellenbosch University, Cape Town, South Africa; South African Centre for Epidemiological Modelling and Analysis (SACEMA), Centre for Epidemic Response and Innovation (CERI), School for Data Science and Computational Thinking, Stellenbosch University, Stellenbosch, South Africa; South African Centre for Epidemiological Modelling and Analysis (SACEMA), Centre for Epidemic Response and Innovation (CERI), School for Data Science and Computational Thinking, Stellenbosch University, Stellenbosch, South Africa; Department of Global Health, Division of Epidemiology and Biostatistics, Faculty of Medicine and Health Sciences, Stellenbosch University, Cape Town, South Africa; South African Centre for Epidemiological Modelling and Analysis (SACEMA), Centre for Epidemic Response and Innovation (CERI), School for Data Science and Computational Thinking, Stellenbosch University, Stellenbosch, South Africa; South African Centre for Epidemiological Modelling and Analysis (SACEMA), Centre for Epidemic Response and Innovation (CERI), School for Data Science and Computational Thinking, Stellenbosch University, Stellenbosch, South Africa; South African Centre for Epidemiological Modelling and Analysis (SACEMA), Centre for Epidemic Response and Innovation (CERI), School for Data Science and Computational Thinking, Stellenbosch University, Stellenbosch, South Africa; Africa Health Research Institute, Durban, South Africa; Institute of Social and Preventive Medicine, University of Bern, Bern, Switzerland; Graduate School for Health Sciences, University of Bern, Bern, Switzerland; Department of Global Health, Division of Epidemiology and Biostatistics, Faculty of Medicine and Health Sciences, Stellenbosch University, Cape Town, South Africa; Lincoln Institute for Rural and Coastal Health, College of Health and Science, University of Lincoln, Lincoln, UK; KwaZulu-Natal Research and Innovation Sequencing Platform (KRISP), University of KwaZulu-Natal, Durban, KwaZulu-Natal, South Africa; South African Centre for Epidemiological Modelling and Analysis (SACEMA), Centre for Epidemic Response and Innovation (CERI), School for Data Science and Computational Thinking, Stellenbosch University, Stellenbosch, South Africa; Department of Global Health, Division of Epidemiology and Biostatistics, Faculty of Medicine and Health Sciences, Stellenbosch University, Cape Town, South Africa; Africa Health Research Institute, Durban, South Africa

**Keywords:** Dolutegravir, HIV, KwaZulu-Natal, South Africa

## Abstract

**Background:**

There is limited evidence on how the rollout of dolutegravir (DTG) has affected retention in care, tuberculosis (TB) disease risk, and viral load suppression (VLS) among people living with HIV (PLHIV) in routine program settings. This study evaluated associations between DTG rollout and VLS, risk of developing TB disease, and retention in care in rural KwaZulu-Natal (KZN), South Africa.

**Methods:**

We employed a retrospective cohort study of PLHIV aged 15 and above, followed up from 1 October 2019 to 31 December 2023 in a rural sub-district of KZN. We grouped antiretroviral therapy (ART) regimens into DTG-containing and non-DTG-containing regimens. We classified PLHIV as virally suppressed or non-suppressed based on a VLS threshold of <400 copies/mL. We used Kaplan-Meier survival curves to describe the transition to DTG over time. We applied Cox proportional-hazards models to evaluate associations between DTG rollout and VLS, the risk of developing TB, and retention in care.

**Results:**

Of the 69 919 PLHIV included in the DTG rollout cohort, approximately 70% (n = 48 598) transitioned to DTG-containing regimens during the 4-year follow-up period. Compared with non-DTG regimens, DTG use was associated with a greater likelihood of VLS (aHR 1.24, 95% CI 1.10–1.29), retention in care (aHR 1.20, 95% CI 1.11–1.30), and lower risk of TB disease (aHR 0.68, 95% CI .54–.87).

**Conclusions:**

These findings support the sustained rollout of DTG-based regimens and emphasize the importance of continuous monitoring to assess their long-term associations and programmatic performance in comparable settings.

The HIV-1 epidemic remains one of the major public health threats in rural KwaZulu-Natal (KZN), South Africa, where HIV incidence is approximately 2.5 per 100 person-years in females and 1.5 per 100 person-years in males aged 15–49 years [1]. Efavirenz (EFV), a non-nucleoside reverse transcriptase inhibitor (NNRTI), has been the main component of the ART since the beginning of the ART program in South Africa in 2004 [2]. The success of EFV-containing ART regimens was threatened by the NNRTI drug-resistant viruses [3, 4], consequently, the World Health Organization (WHO) recommended DTG, an integrase strand transfer inhibitor [5], as an alternative to EFV in 2018 due to its high genetic barrier to resistance [3, 4]. The DTG-containing regimens have been shown to offer many benefits, such as reducing resistance to mutations, minimal risk of side effects, and high drug tolerance when taken correctly and consistently [6–9]. Minor adverse effects associated with DTG use include, but are not limited to, neuropsychiatric effects [10], headache, diarrhea, nausea, insomnia, and fatigue [11]. Evidence on early DTG use has indicated that it was associated with better retention at 12 months [12], has improved viral load suppression (VLS) [13], and is more cost-effective [14] compared with EFV-based ART regimens.

The transition to DTG in South Africa started in September 2019 by initiating newly diagnosed HIV individuals on DTG [3], followed by existing people living with HIV (PLHIV) on NNRTI and protease inhibitors ART regimens. The transition lagged in pregnant women due to a possible association between DTG and neural tube defects in the first trimester [12]. Owing to its favorable safety and tolerability profile, DTG may indirectly contribute to improved adherence and retention in care among PLHIV, as individuals are more likely to remain on therapy that is well tolerated and associated with fewer adverse effects. Given the high HIV burden in South Africa, understanding the long-term impact of DTG-based regimens on treatment success and retention in care is essential for sustaining epidemic control.

Despite the widespread adoption of DTG-based regimens, important evidence gaps persist concerning the overall transition and its long-term associations with VLS, retention in care, and rissk of TB disease , particularly when assessed through routine programmatic data. Understanding how the introduction of DTG-containing regimens has altered patterns of retention in care among PLHIV remains an area requiring further investigation. Evidence on the association between DTG use and TB incidence among PLHIV remains limited. Although DTG has demonstrated superior VLS and immune recovery compared with earlier regimens, it is not yet clear whether these benefits translate into a measurable reduction in TB risk, the leading cause of morbidity and mortality among PLHIV, particularly in high-burden settings such as sub-Saharan Africa. Further evaluation is needed to evaluate the potential indirect effects of DTG rollout on TB prevention and to understand how improved virologic control and retention in care may influence long-term TB outcomes. The underlying hypothesis for the association between DTG use and TB risk was that improved VLS achieved with DTG-based ART may reduce the risk of developing TB disease.

In this study, we present the findings of an evaluation of the long-term associations between the rollout of DTG and VLS, risk of developing TB, and retention in care using data from a high HIV burden setting in rural KZN, South Africa.

## METHODS

### Study Design and Setting.

This study used a retrospective cohort design based on routinely collected clinical and demographic data from PLHIV in the Hlabisa subdistrict of Umkhanyakude, rural KZN, South Africa. The HIV epidemic in Umkhanyakude is one of the world's worst, with an estimated adult HIV prevalence of 30% [15]. The area is nested in a health and demographic surveillance site that has been managed by the Africa Health Research Institute (AHRI) since the year 2000.

### Data Sources

The study employed participant data from the 3 interlinked electronic registers (TIER.NET), an electronic HIV data management system for monitoring and evaluating HIV care in South Africa [16]. TIER.NET was introduced in South African clinics in 2010. PLHIV who started ART before the introduction of TIER.NET had their data transferred into TIER.NET from the previous ART data management system.

### Study Population and Inclusion Criteria

**Figure 1.**
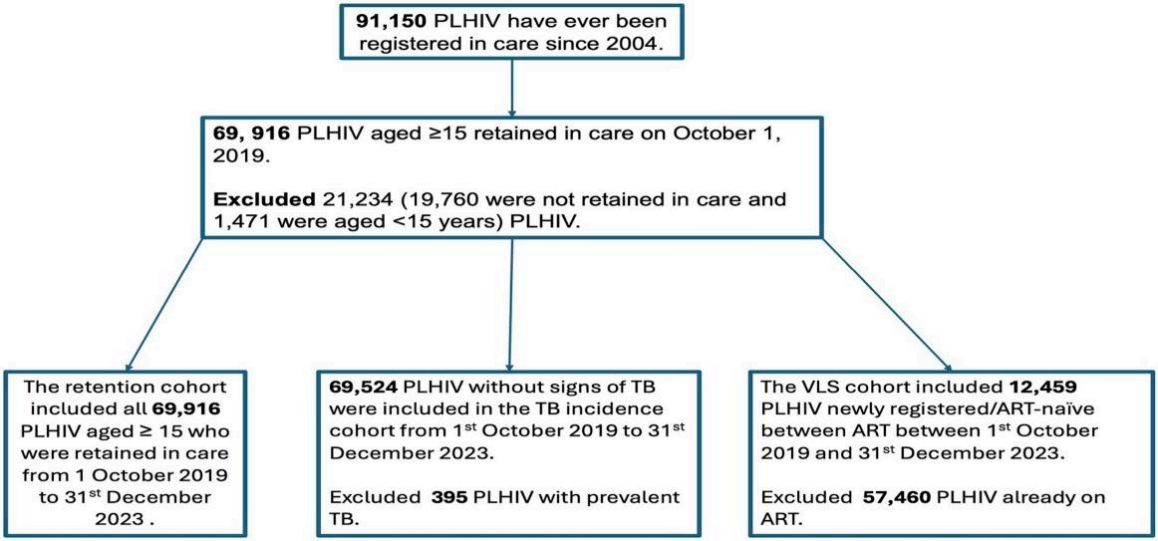
Sample size flow diagram.

We considered PLHIV from 19 clinics within the catchment population. Since the onset of the epidemic and HIV care, a total of 91 150 PLHIV have ever registered in the Hlabisa HIV care program. This study included 69 919 PLHIV aged 15 and above who were still in care between 1 October 2019 and 31 December 2023. The sample sizes and their inclusion and exclusion criteria for each cohort used in the analysis are presented in Figure 1. Children living with HIV aged 14 and below were not included because there were limited pediatric formulations of DTG during the early transition.

### Measures

The following clinical and demographic variables were in our analysis:

1. *Regimen type*: This variable described whether an individual was on a DTG-containing regimen or a non-DTG-containing regimen. All drugs with a DTG component were grouped into the DTG-containing regimen, and those without a DTG component were grouped into the non-DTG-containing group.
2. *VLS*: VLS was created from the viral load variable in the dataset. Viral load is routinely monitored among PLHIV in care to monitor progress. This time-varying variable showed whether an individual is virally suppressed or not at different follow-up points. Participants with ≤400 copies per milliliter of blood were considered virally suppressed, while those with >401 were considered virally unsuppressed. A viral load cutoff of 400 copies/mL was used to define VLS in this study because the threshold aligns with historical and programmatic definitions used in South Africa and other resource-limited settings, where earlier generations of viral load assays had lower limits of detection around 400 copies/mL [17]. Using this cutoff ensures consistency and comparability with data from earlier cohorts and national HIV program indicators [18, 19].
3. *TB disease*: This time-varying variable described PLHIV with TB disease. This variable was derived from the TB status variable in TIER.NET. Detection of TB follows the WHO-recommended programmatic approach to TB screening using the 4-symptom screen for PLHIV and the general population, which includes cough of any duration, fever, night sweats, and unexplained weight loss [20]. A positive symptom screen subsequently triggered further diagnostic evaluation, including sputum-based molecular testing and chest X-ray imaging, which enabled confirmation of active TB disease among a subset of symptomatic individuals. In addition, certain priority groups, such as newly diagnosed PLHIV and pregnant women, undergo TB diagnostic testing routinely, regardless of the presence or absence of symptoms, in accordance with national and WHO recommendations.
4. *Retention in care*: This variable was created from the outcome variable in the TIER.NET dataset. PLHIV who did not show up in clinics for 3 consecutive months (90 days) and those who died were grouped in 1 category as not retained. We grouped PLHIV who died and those lost to follow-up because HIV deaths are not well captured in TIER.NET, often misclassified as loss to follow-up [21].
5. *CD4 count categories*: This variable was created from the CD4 count variable in TIER.NET. CD4 count is a measure of the person's immune system, and therefore the stage of disease. CD4 in cells/mm^3^ was categorized as follows: 0–199, 200–499, 500–999, and above 1000. CD4 was a time-varying measure in the dataset.
6. *Sex of participants*: This variable described the biological differentiation of sex into males and females as assigned at birth.
7. *Participant's age groups*: Age groups were generated from the participants' age variable, generated by subtracting the observation date from the participant's date of birth, to create age at different observation dates, which were then categorized. Participants' age was categorized into the following categories: 15–24, 25–34, 35–44, 45–54, and above 55 years.

### Data Analysis

All analyses were conducted using StataCorp statistical package version 18 [22]. In our DTG scale-up analysis cohort, we retrospectively followed up 69 916 PLHIV who were still in care as of 1 October 2019, excluding those who were lost to follow-up and died before 1 October 2019. The effect of DTG on the development of TB disease included 69 524 PLHIV without TB disease at the start of the follow-up. Different cohorts used in the analysis have been presented in detail in Figure 1. We included newly registered PLHIV in our VLS cohort to avoid bias associated with including PLHIV who were already on ART and who may have been suppressed already. Missing CD4 and viral load data were handled using multiple imputation.

Kaplan-Meier survival curves were used to describe transition to DTG over time overall, and by key subgroups: sex, age, TB status, and CD4 count. Since data came from 19 clinics that may have influenced the transition of PLHIV to DTG-containing regimens, we applied random-effect Cox proportional hazard models to individually model associations between DTG rollout and VLS, risk of developing TB disease, and retention in care. The Cox proportional hazard models are distribution-free models that assume that the survival/failure curves for 2 or more strata of the predictor variables are proportional over time [23]. The effect of DTG on retention in the care model was controlled for sex, age, years on ART, CD4 count, and TB status as confounders. The effect of DTG on the VLS model was controlled for sex, age, years on ART, CD4 count, and TB status. The effect of DTG on the risk of developing TB model was controlled for sex, age, VL, and CD4 count. Time-varying covariates included in the models were age, TB status, VLS, and CD4 count. We confirmed that the proportional hazard assumption was met for all the models.

## RESULTS

**Figure 2.**
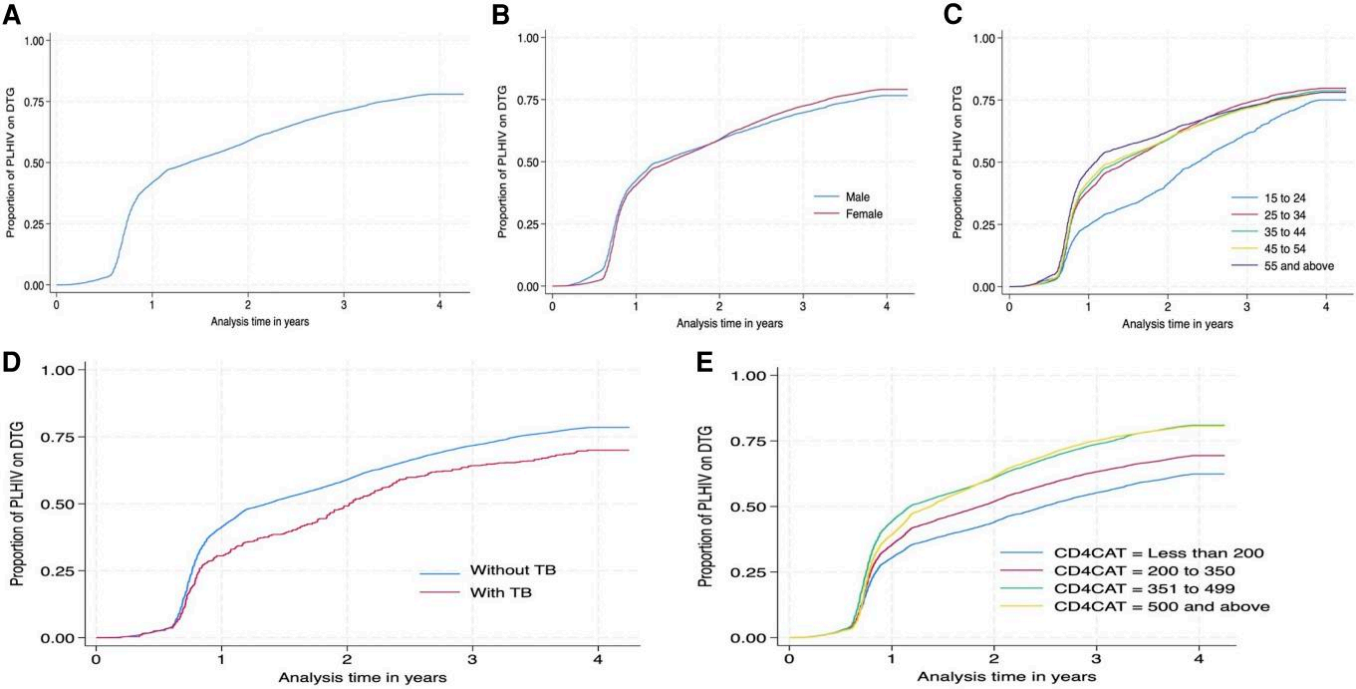
Overall proportion of PLHIV transitioning to DTG (a), and by sex (b), age groups (c), TB disease status (d), and CD4 count (e).

At the start of follow-up, 58 807 PLHIV (41 283 females and 17 524 males) were included in the retention-in-care cohort. An additional 11 112 individuals entered the cohort over the course of the study period (Table 1). The proportion of PLHIV who were on DTG-containing regimens at the beginning of follow-up was 1.5%. However, by 31 December 2023, approximately 70% (48 598) of the 69 916 individuals in the rollout cohort had transitioned to a DTG-containing regimen (Supplementary Table 1). The overall proportion of PLHIV transitioning to DTG-based regimens is shown in Figure 2*A*. In the early phase of the rollout, a smaller proportion of females transitioned to DTG than males; however, from 2021 onward, uptake among females surpassed that of males (Figure 2*B*). As illustrated in Figure 2*C*, DTG uptake increased steadily across all age groups, though it remained notably lower among individuals aged 15–24 years. Throughout the analysis period, PLHIV with TB were consistently less likely to transition to DTG than those without TB (Figure 2*D*). Similarly, those with low CD4 counts showed persistently lower transition rates over time (Figure 2*E*).

**Table 1.** Description of Baseline Characteristics of the PLHIV (N = 58 807)

Characteristic	Frequency	Column %
Sex		
Male	17 524	29.8
Female	41 283	70.2
Age group		
15–24	3035	5.2
25–34	12 148	20.7
35–44	20 353	34.6
45–54	13 436	22.9
55 and above	9835	16.6
CD4 count categories		
<200	8533	14.5
200–350	10 932	18.6
351–499	11 950	20.2
500 and above	27 392	46.8
Viral load suppression		
No	21 112	35.9
Yes	37 695	64.1
TB disease		
Symptomatic	941	1.6
Asymptomatic	57 866	98.4
Regimen type		
DTG containing	882	1.5
Non-DTG containing	57 925	98.5

Baseline characteristics were based on the PLHIV who were still in care at the beginning of the follow-up on 1st October 2019. 58 807 PLHIV were available at the beginning of follow-up, while 11 112 PLHIV started ART during the follow-up period.

Results from the multivariable Cox proportional hazards models (Table 2) indicated that the likelihood of VLS was 24% higher among PLHIV who transitioned to DTG-containing regimens compared with those on non-DTG regimens (aHR 1.24, 95%CI 1.10–1.29). Conversely, the likelihood of VLS was 15% lower among PLHIV who developed TB compared with those without TB (aHR 0.85, 95% CI .75–.97). The likelihood of VLS was 32% higher in PLHIV with CD4 counts between 351 and 499 cells/μL (aHR of 1.32, 95% CI: 1.11–1.52), and 62% higher in PLHIV with CD4 ≥ 500 cells/μL (aHR of 1.62, 95% CI 1.27–1.97) compared with PLHIV with CD4 ≤ 350 cells/μL. In contrast, the likelihood of VLS was 19% lower among PLHIV aged 35–44 years (aHR = 0.81, 95% CI .76–.86) and 20% lower among those aged ≥55 years (aHR 0.80, 95% CI .75–.85), compared with those aged 15–24 years.

**Table 2.** Multivariable Cox Proportional Hazard Models of the Effect of Dolutegravir on Viral Load Suppression, Retention in Care, HIV Mortality, and Development of TB disease

	Retention in Care	Viral Load Suppression	Risk of TB Disease
	AHR (95%CI)	AHR (95%CI)	AHR (95%CI)
Regimen type			
EFV	1	1	1
DTG	1.20(1.11, 1.30)**	1.24 (1.10,1.29)***	.68 (.54, .87)**
Sex			
Male	1	1	1
Female	.88(.81,.97)	1.03 (.99,1.09)	.42(.34, .53)***
Age groups			
15–24	1	1	1
25–34	.91(.80, 1.04)	1.53(1.45,1.62)***	.91(.60, 1.39)
35–44	.67(.59,.78)***	1.15(1.09,1.21)***	.98(.65, 1.47)**
45–54	.49 (.41, .58)***	.81(.76,.86)***	.72(.46, 1.15)
≥55	0.54(.43, .68)***	.80 (.75, .85)***	.76 (.45, 1.28)
CD4 count			
0–199	1	1	1
200–350	1.01 (.83, 1.24)	1.12(.83, 1.52)	.40 (.26, .64)
351–499	.88 (.77, 1.05)	1.32 (1.11, 1.52)**	.35 (.26, .46)
500–999	.93(.78,1.11)	1.62 (1.27 1.97)**	.22 (.13, .36)*
On TB treatment			
No	1	1	…
Yes	1.44(1.20,1.72)	.77(.64,.92)**	—
Virally suppressed			
No	1	-	…
Yes	1.27 (1.11, 1.43)	-	.91 (.65, 1.15)

*Note on multivariate Cox regression models*: The effect of DTG on retention was adjusted for sex, age, years on ART, CD4 count, viral load, and TB status. The effect of DTG on VLS model was adjusted for sex, age, years on ART, CD4 count, and TB status. The effect of DTG on the risk of developing TB model was controlled for sex, age, viral load, and CD4 count.

Abbreviation: AHR, adjusted hazard ratios.

***P < .001, **P < .005, *P < .05.

DTG use was associated with a 20% increase in the likelihood of retention in care (aHR 1.20, 95% CI 1.11–1.30) compared with other regimens. The likelihood of being retained in care was slightly lower in PLHIV with higher CD4 counts (350–499 and ≥500 cells/μL) compared with those in lower CD4 categories. The likelihood of retention in care was also lower among PLHIV aged 35–44 years (aHR 0.67, 95% CI .59–.78) and those aged 45–54 years (aHR 0.49, 95% CI .41–.58), relative to PLHIV aged 15–24 years. In contrast, PLHIV who were virally suppressed had a higher likelihood of being retained in care compared with those who were not suppressed (aHR 1.27, 95% CI 1.11–1.43). Similarly, PLHIV who developed TB disease were associated with a higher likelihood of being retained in care compared with those without TB disease (aHR 1.44, 95% CI 1.20–1.72).

The risk of developing TB was 32% lower among PLHIV on DTG compared with their counterparts on non-DTG containing regimens (aHR 0.68, 95% CI .54–.87). The risk of developing TB was 58% lower in women compared with their male counterparts (aHR 0.42, 95% CI .34–.53). TB disease was less likely among PLHIV with higher CD4 counts, the risk of developing TB among PLHIV with CD4 count above 500 cells/mm^3^ were 78% lower (aHR 0.22, 95% CI .13–.36) while the risk of developing TB among PLHIV with CD4 count between 351 and 499 were 65% lower (aHR 0.35, 95% CI .26–.46) compared with those with CD4 count less than 200 cells/mm^3^.

**Figure 3.**
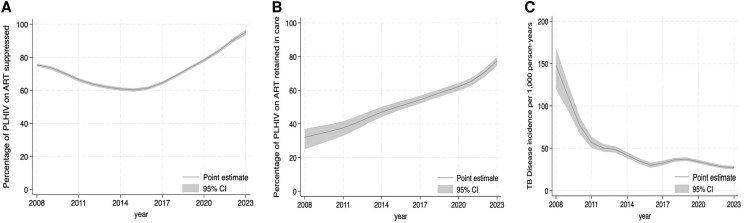
Longitudinal trends in VLS (a), retention in care (b), and TB disease incidence (c) among PLHIV.

Temporal trends in VLS and retention in care demonstrated consistent improvements over the study period. As illustrated in Figure 3*A*, the proportion of individuals achieving VLS increased steadily from 63% in 2019 to 92% in 2023. Similarly, retention in care improved during the same period (Figure 3*B*), from 68% in 2019 to 80% in 2023. The incidence of TB among PLHIV showed a substantial decline, decreasing from 50 per 1000 person-years in 2013 to 27 per 1000 person-years in 2023 (Figure 3*C*). This downward trend in TB incidence likely reflects both improved immune restoration due to higher VLS rates and expanded access to preventive TB interventions within HIV programs.

## DISCUSSION

This study evaluated the long-term associations between the scale-up of DTG and VLS, retention in care, and the risk of developing TB in rural KZN, South Africa. Insights from the data demonstrate that the transition to DTG was associated with a higher likelihood of VLS, retention in care, and reduction in the risk of developing TB among PLHIV over a 4-year follow-up period.

The low transition to DTG observed in PLHIV coinfected with TB could be attributed to fears of drug-drug interactions with Rifampicin [24], a key antimicrobial agent used as a core component of combination therapy for TB [25]. The current HIV/TB guidelines recommend that PLHIV co-infected with TB who are treated with rifampicin receive a doubled dose of DTG [26]. The delayed switch to DTG could also be related to the higher rates of TB seen among people not using DTG because weaker viral suppression and slower CD4 recovery may increase their risk of developing TB. Health care providers' decisions about which patients to transition to DTG based on patients' ART history, comorbidities, and treatment response before initiating DTG-based regimens may have influenced delays in the overall transition among certain patient groups, particularly those with prior treatment failure, potential drug–drug interactions, or contraindications, resulting in a more gradual and selective implementation of DTG across the program [27].

The positive association between DTG and clinical outcomes demonstrated in this study implies that transitioning all PLHIV to DTG could increase VLS, retention in care and decrease the incidence of TB disease in PLHIV, provided resistance to the current DTG-containing regimen is minimal. Progress in VLS may help reduce new HIV infections, as PLHIV who achieve and maintain suppression are much less likely to transmit the virus [28, 29]. Our findings are consistent with related studies that found a high likelihood of VLS in PLHIV on DTG-containing regimens [3, 12] in South Africa.

The rollout of DTG may have strengthened retention in care among PLHIV due to its superior efficacy, favorable safety profile, and simplified dosing compared with older antiretroviral therapies [11]. Dolutegravir-based treatments generally cause fewer adverse effects, achieve faster viral suppression, and have a stronger resistance barrier, which collectively promote better adherence and patient satisfaction [11, 30]. As treatment becomes more tolerable and effective, individuals are more likely to stay engaged in HIV care and adhere to their medication, ultimately supporting sustained viral control and improved retention within treatment programs. Our findings further suggest that control of the AIDS pandemic as a public health threat could be achieved if measures to strengthen retention in care are put in place through interventions such as defaulter tracing and integration of HIV care in community clinics [31]. Despite the improvements in clinical outcomes demonstrated in this study, HIV drug resistance in ART-experienced patients in rural KZN post-DTG rollout poses a major threat to the success of the DTG rollout [32]. Increasing cases of resistance to the DTG regimen [33], suggest that further evaluation and monitoring of the long-term performance of DTG are required.

Based on these findings, we recommend that health care workers intensify screening to identify groups of PLHIV not yet transitioned to DTG in health care facilities. We also recommend that all clinic follow the South African ART guidelines on the management of HIV/TB coinfection; these guidelines have been made available in all clinics providing HIV services. Measures be put in place to ensure that HIV services are not disrupted during future pandemics to sustain gains in clinical outcomes. Lastly, there is a need for engaging PLHIV with high viral loads to improve adherence and VLS rates through care groups and other peer networks.

A major strength of this study is that it provides one of the few large-scale, real-world evaluations of DTG rollout in routine care within a resource-limited and high HIV prevalence, rural African setting. Also, in this article, we evaluated the long-term associations between DTG and VLS, retention in care, and risk of TB disease, instead of looking at each outcome in a separate analysis. Evaluating programmatic outcomes beyond the controlled environment of clinical trials provides crucial evidence on the effectiveness, feasibility, and challenges of implementing DTG-based regimens in everyday practice. The findings not only complement results from randomized studies but also offer practical insights to inform national treatment strategies and optimize antiretroviral therapy delivery. Importantly, the study's results have direct implications for accelerating progress toward South Africa's and UNAIDS' 95-95-95 targets by improving VLS rates and strengthening health system performance.

This study has limitations. First, it was conducted in 19 clinics within the Hlabisa health sub-district, an area with a high HIV prevalence, and therefore, the generalizability of our findings may be limited in settings with lower HIV prevalence or differing patterns of DTG rollout, where implementation occurred more slowly and less uniformly. Nonetheless, findings from this high-burden district provide valuable insights into programmatic performance in similar settings. Secondly, potential misclassification of outcomes may have biased our estimates of the association between DTG use and clinical outcomes,for example, deaths misclassified as loss to follow-up, or vice versa. Thirdly, loss to follow-up among participants represents another possible source of bias, particularly if differential attrition occurred between groups. The fourth limitation relates to clinician decision-making which may have influenced who was initiated on DTG, especially if prescribing practices deviated from national guidelines, introducing potential selection bias. Fifth, residual confounding may have affected the observed associations between DTG use and outcomes such as VLS, retention in care, and risk of TB, particularly if relevant variables influencing these outcomes were not controlled for in our Cox proportional hazards models. Finally, disruptions in service delivery during the COVID-19 pandemic may have affected the completeness and quality of data collected before and after the onset of the pandemic, which could in turn have influenced our findings.

### Conclusion

Our analysis demonstrates that the rollout of DTG has contributed to HIV epidemic control by improving VLS, reducing the risk of TB, and enhancing retention in care among PLHIV. Broader transition of all PLHIV to DTG-based regimens, consistent with WHO recommendations, coupled with sustained engagement in care, could yield long-term benefits for individual clinical outcomes and further reduce HIV transmission at the population level.

## Supplementary Material

ofag156_Supplementary_Data

## Supplementary Data

Supplementary materials are available at *Open Forum Infectious Diseases* online. Consisting of data provided by the authors to benefit the reader, the posted materials are not copyedited and are the sole responsibility of the authors, so questions or comments should be addressed to the corresponding author.
