# Supplementary material for: Effect of the Scale-Up of Dolutegravir on Retention in Care, Risk of Developing Tuberculosis and Viral Load Suppression Among People Living With HIV: Analysis of Routine HIV Clinical Data in Rural KwaZulu-Natal, South Africa (2019–23)
Source: Open Forum Infect Dis. 2026 Mar 19;13(4):ofag156. doi: 10.1093/ofid/ofag156 (PMC13059684; doi:10.1093/ofid/ofag156)
Supplement: ofag156_Supplementary_Data [file ofag156_supplementary_data.docx]

Supplementary Table 1: Description of participants' characteristics and their person-years lived in each category

| **Characteristic** | **Person-years lived in the category** | **Percentage** |
| --- | --- | --- |
| **Age group** |  |  |
| 15 – 24 | 24,412 | 6.1 |
| 25 – 34 | 69,572 | 17.3 |
| 35 – 44 | 127,998 | 31.7 |
| 45 – 54 | 100,941 | 25.0 |
| 55 and above | 80,304 | 19.9 |
| **Sex** |  |  |
| Male | 115,229 | 28.6 |
| Female | 287,890 | 71.4 |
| **Viral load suppression** |  |  |
| No | 113,026 | 28.1 |
| Yes | 290,201 | 71.9 |
| **CD4 categories** |  |  |
| <200 | 30,719 | 7.6 |
| 200 to 350 | 38,656 | 9.6 |
| 351 to 499 | 237,455 | 58.9 |
| 500 and above | 96,397 | 23.9 |
| **Tb disease symptoms** |  |  |
| Symptomatic | 4,888 | 1.2 |
| Asymptomatic | 398,339 | 98.8 |
| **Regimen type** |  |  |
| DTG containing | 319,698 | 79.7 |
| Non-DTG containing | 83,421 | 20.3 |
|  |  |  |

We used person-years lived in each category because the data were longitudinal, resulting from multiple visits. The percentage indicates the proportion of time an individual spent in that particular disease category.
